# The encapsulation effect of UV molecular absorbers into biocompatible lipid nanoparticles

**DOI:** 10.1186/1556-276X-6-73

**Published:** 2011-01-12

**Authors:** Ioana Lacatusu, Nicoleta Badea, Alina Murariu, Aurelia Meghea

**Affiliations:** 1 Faculty of Applied Chemistry and Materials Science, University POLITEHNICA of Bucharest, Polizu Street No. 1, 011061 Bucharest, Romania

## Abstract

The efficiency of a cosmetic product depends not only on the active ingredients, but also on the carrier system devoted to improve its bioavailability. This article aims to encapsulate two couples of UV molecular absorbers, with a blocking action on both UV-A and UV-B domains, into efficient lipid nanoparticles. The effect of encapsulation on the specific properties such as sun protection factor and photostability behaviour has been demonstrated. The lipid nanoparticles with size range 30-350 nm and a polydispersity index between 0.217 and 0.244 are obtained using a modified high shear homogenisation method. The nanoparticles had spherical shapes with a single crystallisation form of lipid matrices characteristic for the least ordered crystal structure (α-form). The *in vitro *determination of photoprotection has led to high SPF ratings, with values of about 20, which assure a good photoprotection and filtering about 95% of UV radiation. The photoprotection effect after irradiation stage was observed to be increased more than twice compared to initial samples as a result of isomerisation phenomena. All the results have shown that good photoprotection effect and improved photostability could be obtained using such sunscreen couples, thus demonstrating that UV absorbers-solid lipid nanoparticles are promising carriers for cosmetic formulations.

## Introduction

The methodologies for nanoparticles synthesis represent a promising approach which may be used to develop new biocompatible carrier systems for various compounds with lipophil character such as UV chemical absorbers and applications in cosmetic field. The dermato-cosmetic products with photoprotective effect have represented and continue to represent a real challenge for cosmetic industry.

The protection against UV radiation became a prominent problem for human health because of harmful effects of UV radiation on skin such as: skin drying, spots emergence, erythema, rapid ageing of skin (wrinkles, photoageing) and induction of skin cancer [1]. Photoprotection is an essential prophylactic and therapeutic element which is very important in order to avoid all these undesirable effects [2]. The most reliable indicator for evaluating the photoprotection degree is the sun protection factor (SPF) rating. The SPF corresponds to the multiple of time during which the sunscreen will prevent obvious reddening of the skin, over the exposure time that causes unprotected skin to exhibit reddening.

The substances with SPF have been widely used as photoprotective agents for a long time in the cosmetic industry, but their encapsulation in biocompatible lipid nanoparticles with enhanced properties was not fully elucidated; only a few publications for this research area being presented in the literature [3,4]. As a result, the solar protection agent formulation, which aimed at improving UV protective effect, is a subject of great importance in order to avoid exposure to harmful ultraviolet radiation and the response injury induced by UV photons in skin [5], simultaneously with minimising of local adverse effects.

The efficiency of a cosmetic product depends not only on the active ingredients, but also on the carrier system with the aim to improve its bioavailability. The real efficacy of new or old active compounds is not enough for obtaining a cosmetic product really efficient. The product depends not only on used active principles, but also on the penetrating degree into the skin layers which is strongly dependent on the used carriers. The nanodisperse systems represent a mild way in order to enhance the penetration degree and increase the performance of a cosmetic product [6,7]. In this context, lipid nanoparticles are attractive colloidal carrier systems for cosmetics and dermatologic formulations due to their beneficial effects on skin, compared to other colloidal carrier systems [7], being based on nontoxic and nonirritant lipids [8]. This is the most remarkable advantage of these systems - the lipid matrix being composed of well-tolerated and physiological lipids, thus leading to minimise the danger of acute and chronic toxicity.

Due to the lipid biocompatibility, the self-assembling capacity, versatility of the particle size and low cost, systems based on lipid nanoparticles have become the subject of many topics of research, most of them developed by Müller [who discovered solid lipid nanoparticles (SLN) systems in 1991 and later the nanostructured lipid carriers systems, 1999] for cosmetics formulations used mainly for local treatment of skin diseases [9,10].

After 2005 the lipid nanoparticles systems have gained attention in a continuous growth amongst researchers in cosmetic sector due to their ability to prevent the deficiencies of both systems existent up to their occurrence: microcapsules and classic colloidal delivery systems [11,12]. The lipid nanoparticles systems present some features and in the same time advantages that recommend them as promising carrier systems for cosmetic applications [13]: provide an improved stability of chemical labile active ingredients [14]; are able to provide a carrier system with controlled release [15]; show occlusive properties which help in formation of film on skin [15]; present a high potential to block UV radiation [16].

The use of lipid nanoparticles as a new generation of carrier systems for UV absorbers has been introduced only a few years ago [17]. It was shown that these lipid nanoparticles present a high potential to inhibit the UV radiation, they may act as a specific physical UV sunscreen by efficient scattering of light, being thus able to improve the sun protection effect [11]. The first article that has opened the development of distribution systems based on lipid nanoparticles for UV absorbers was drawn up by Müller in 2002 [18]. The improved efficiency of lipid carrier, based on *in vitro *investigations, was demonstrated by encapsulation of a classic sunscreen - 3-*benzophenone *in crystalline lipid nanoparticles. Similarly, Wissing and Muller [13] have conducted several *in vitro *release studies of another lipophil sunscreen widely used in cosmetic formulations - *oxybenzone*. The preparation and characterisation of SLNs with *cetyl palmitate *loaded with an absorber with broad spectrum of action on both UV-A and UV-B domain (*Ethylhexyloxyphenol methoxyphenyl triazine*), was described in a research published three years ago [4].

Therefore, this investigation will focus on the study of the behaviour of two couples of UV molecular absorbers, two of the constituents having a blocking action on UV-B (2-ethylhexyl-2-cyano-3,3-diphenylacrylate, OCT and 2-ethylhexyl trans-4-methoxycinnamate, OMC) and one manifesting a broad action on both UV-A and UV-B domains (*Bis*-ethylhexyloxyphenol methoxyphenyl triazine, BEMT), after encapsulation into efficient lipid nanoparticles. Moreover, their specific properties: photoprotective index and photostability behaviour, have been characterised. Finally, for exploring the potential of SLNs in improving the photostability in mild irradiation conditions, some cosmetic formulations were developed and evaluated, based on a combination between a cream base with OMC-OCT - SLN and BEMT-OCT - SLN.

## Experimental

### Materials

Polyethylene glycol sorbitan monooleate (Tween 80) was purchased from Merck (Germany); Synperonic PE/F68 (block copolymer of polyethylene and polypropylene glycol), L-α-Phosphatidylcholine (Lecithin), OCT, 97% and OMC, 98% were obtained from Sigma Aldrich Chemie GmbH (Munich, Germany); *n*-hexadecyl palmitate (CP), 95% was purchased from Acros Organics (USA); glyceryl stearate (GS), *Bis*-BEMT and the cream base (which contains stearates, glycerine, fatty alcohols, emulsifier, emollients and an antioxidant - butylhydroxyanisole) were supplied by Elmiplant S.A. Company, Romania.

### Synthesis of sunscreen nanoparticles embedded into lipid matrices

**Figure 1:**
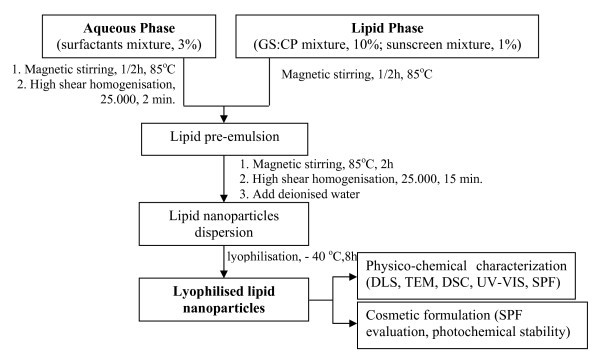
**Synthesis procedure of some couples of UV molecular chemical absorbers encapsulated into lipid nanoparticles**.

Different GS:CP nanosuspensions were produced by a modified melt homogenisation method. The steps followed in synthesis of lipid nanoparticles loaded with both couples of molecular sunscreens (OMC-OCT-SLN and BEMT-OCT-SLN) are presented in Figure 1. The lipid mixture (hexadecyl palmitate:GS = 1:1, w/w) was melted at the temperature of 85°C. In the melted lipids that represent 10% from the total SLN dispersion, an amount of 1% sunscreen mixture was added. A solution of polyethylene glycol sorbitan monooleate, synperonic PE and lecithin (1:0.25:0.25, w/w) in deionised water was heated to the same temperature. Before the forming of lipid pre-emulsion, the aqueous surfactant solution was processed by high shear homogenisation (using a Lab High-Shear Homogeniser SAII-20 type; 0-28,000 rpm and power of 300 W, Shanghai Sower Mechanical & Electrical Equipment Co., Ltd., China) for 2 min at 25,000 rpm in order to destroy the multilamellar liposome formed by lecithin. The hot pre-emulsion was further processed by applying 25,000 rpm for 15 min. The lipid nanoparticles dispersion obtained by adding 50 mL water was exposed to lyophilisation in order to increase the loaded-SLN concentration (using a Christ Delta 2-24 KD lyophiliser, Germany). The sunscreen loaded lipid nanoparticles have been analysed by dynamic light scattering (DLS), TEM, DSC, UV-Vis techniques and SPF analyses.

### Methods and equipment for lipid nanoparticle characterisation

#### DLS technique

Particle size (*z*-average) and polydispersity index (PI) of each SLN dispersion were determined after 1 day of preparation and few months later, using dynamic light scattering technique (Zetasizer Nano ZS, Malvern Instruments Ltd., UK), at a scattering angle of 90° and 25°C. Dispersions were analysed after appropriate dilution with deionised water to an adequate scattering intensity prior to the measurement. The particle size analysis data were evaluated using intensity distribution. The zeta potential of the SLN dispersions was evaluated with the same DLS technique. For each sample, the hydrodynamic radius and zeta potential have been measured in triplicate.

#### Transmission electronic microscopy

The morphology of OMC-OCT - SLN and BEMT-OCT - SLN was examined using a transmission electron microscope (Philips 208 S, Netherlands). A drop of the diluted lipid nanoparticle solution was placed onto a carbon-coated copper grid and kept for 15 min before the samples were viewed and photographed.

#### Differential scanning calorimetry

In order to investigate the changes in the crystallinity of the lipid matrix, DSC analysis was performed. Thermograms were recorded with a differential scanning calorimeter Jupiter, STA 449C (from Netzsch Instruments N.A. LLC). Samples were heated at the scanning rate of 3°C/min over a temperature range between 30 and 100°C.

#### In vitro determination of SPF

The determination of SPF ratings was realised using UV-Vis V670 Spectrophotometer equipped with integrated sphere and the adequate soft. For SPF evaluation, an amount of 2 mg/cm^2 ^cream is applied onto Transpore™ 3M support (a synthetic skin) and the sample spectrum is registered on 290-400 nm, using a reference support - Transpore™ 3M without cream. The method for *in vitro *determination of SPF of sunscreens is based on Diffey and Robson theory [19]:

$$\text{SPF}=\frac{\sum {}_{\left(400-290\right)}E_{\lambda }\cdot B_{\lambda }}{\sum {}_{\left(400-290\right)}\frac{E_{\lambda }\cdot B_{\lambda }}{{\text{MPF}}_{\lambda }}}$$

where *E*_λ _sun radiation extinction for Earth (between 20° and 40° N latitude); *B*_*λ *_relative extinction for each wavelength; MPF_λ _the monochromatic protection factor for selected wavelength (the difference between the spectrum of measured sample applied on support and support spectrum).

#### UV-A and UV-B irradiation

The photostability of UV-absorber couples-SLN has been evaluated by irradiation on UVA-UVB with an energy of 19.5 J/cm^2^, at two wavelengths: 365 nm (UVA) and 312 nm (UVB) on a short period (1 h on UVA and 2 h on UVB - *irradiation I*) and prolonged period of time (2 h on UVA and 4 h on UVB - *irradiation II*), using Irradiation System BioSun, Vilver Lourmat, France. The extent of photodegradation was monitored by recording the absorption spectra in the wavelength range 290-400 nm on a UV-Vis V670 Spectrophotometer (Jasco, Japan), using the accessory with integrated sphere.

## Results and discussion

### Size distribution and stability of UV absorbers couples - SLN

The SLNs suspension is a heterogenous system with co-existence of additional colloidal structures (micelles, liposomes, supercooled melts) which caused a specific size distribution [11,20], depending on the selected preparation procedure. For this reason, even in the literature there are some preparation procedures [e.g. high pressure homogenisation (HPH), microemulsion, solvent diffusion, high shear homogenisation coupled with ultrasound technique], the most used technique for production of SLNs is HPH which allows obtaining of a narrow size distribution of nanoparticles.

**Figure 2:**
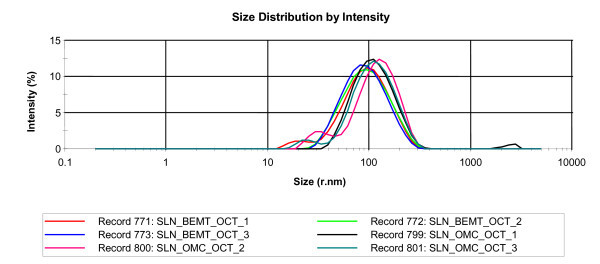
**Size distribution of lipid nanoparticles evaluated by dynamic light scattering**.

In this study is demonstrated the possibility to obtain lipid nanoparticles with relatively narrow size distribution and no micron particles using a modified-HSH technique, without an additional ultrasound treatment. Due to the use of lecithin that is not able to form micelles in aqueous solution, it forms only liposomes, a supplementary shear homogenisation of surfactant aqueous solution has led to expected results. All the nanoparticles formulated in this study were completely distributed in the size range 20-350 nm (Figure 2). The results obtained by DLS evidenced that for both couples of UV absorbers encapsulated into lipid nanoparticles, a relatively narrow size distribution was observed, with a polydispersity ranging between 0.217 and 0.244. The average size of lipid nanoparticles after 1 day of preparation was about 96.5 nm (for OMC-OCT - SLN) and about 79.5 nm (for OCT-BEMT - SLN).

**Figure 3:**
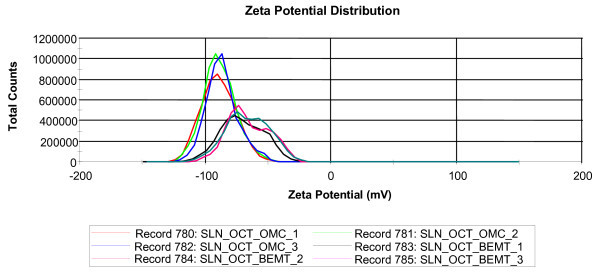
**Zeta potential distribution for OMC-OCT - SLN and EMT-OCT - SLN**.

The measurement of zeta potential allows predictions about the storage stability of colloidal systems. In general, the particles aggregation is unlikely to appear if the particles are charged and present high zeta potential values due to the electrostatic repulsions. The zeta potential distribution for both OMC-OCT - SLN and BEMT-OCT - SLN is shown in Figure 3. The zeta potential values start from -50 mV for OMC-OCT - SLN (with an average potential of -85 mV) and from -25 mV for BEMT-OCT - SLN (with an average potential of -67 mV), respectively. These highly electronegative values demonstrate that using this method a high stability of SLN systems and good size distribution are obtained.

In Table 1 are collected the data of particle size of lipid nanoparticles loaded with molecular UV-absorber after 1 day of preparation and after a few months of storage at 4°C. The SLN suspensions show sufficient long-term stability with only slight particle size increase after storage.

**Table 1:** The size evolution/stability of OMC-OCT - SLN and BEMT-OCT - SLN in time

OCT-OMC - SLN							
***After 24 h***		***After 2 months***		***After 9 months***		***After 12 months***	

**Z _average _[nm]**	**Pdl**	**Z _average _[nm]**	**Pdl**	**Z _average _[nm]**	**Pdl**	**Z _average _[nm]**	**Pdl**

96.0	0.240	94.9	0.271	98.7	0.211	101.6	0.242
94.5	0.240	96.2	0.273	97.4	0.244	100.1	0.233
94.4	0.231	98.5	0.273	98.8	0.228	98.9	0.235

**OCT-BEMT - SLN**							

***After 24 h***		***After 2 months***		***After 9 months***		***After 12 months***	

**Z _average _[nm]**	**Pdl**	**Z _average _[nm]**	**Pdl**	**Z _average _[nm]**	**Pdl**	**Z _average _[nm]**	**Pdl**

79.2	0.244	81.3	0.220	85.4	0.235	88.2	0.203
79.4	0.219	81.9	0.204	84.8	0.218	86.7	0.200
79.7	0.217	81.9	0.216	83.3	0.229	83.2	0.223

### Morphologic and crystalline characteristics of molecular absorbers loaded into SLN

**Figure 4:**
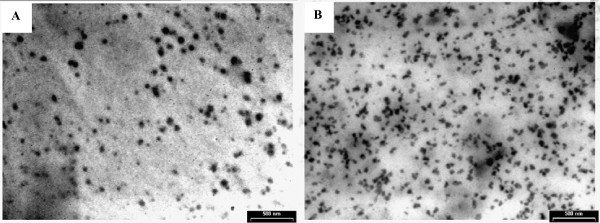
**TEM images of lipid nanoparticles: OCT-OMC - SLN (a) and OCT-BEMT - SLN (b)**.

**Figure 5:**
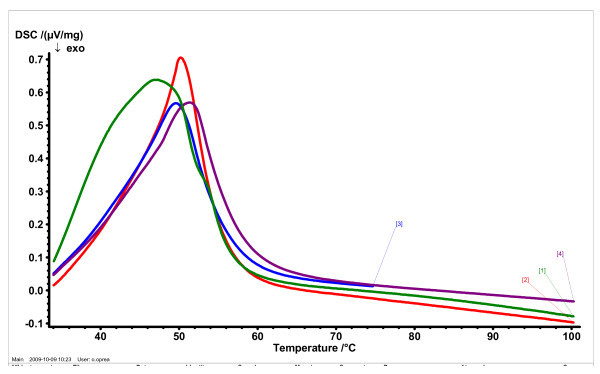
**Thermal behaviour of: (1) bulk lipid matrix; (2) unloaded SLN; (3) SLN loaded with mixture of OMC and OCT; (4) SLN loaded with mixture of BEMT and OCT**.

TEM images of SLN loaded with both couples of UV absorbers which are shown in Figure 4 indicated that the particles had nanometre size and spherical shapes and no irregular crystallisation with the majority of needle crystals visible. This last aspect underlines the higher content in the least ordered crystal structure (α-form) in the lipid phase, whilst the perfect crystals manifest a typical elongated, needle-shaped crystals characteristic to a more ordered structure (β modification) [21,22]. The most stable β form is not desired due to the expulsion in time of UV absorbers. This observation is also confirmed by DSC analysis where there is a single crystallisation form of lipid matrices (Figure 5).

Figure 5 shows the allure of the melting process of bulk lipid matrix (physical mixture of CP and GS), free lipid nanoparticles and lipid nanoparticles loaded with UV absorbers. From DSC curves it is observed that the crystallinity was different in the bulk lipid mixture, free-SLN and loaded-SLN, due to the presence of surfactants and molecular UV absorbers in their compositions. The lipid mixture exhibits a broad melting range, whilst the lipid nanoparticles have a narrow peak at 50.2°C (for empty SLN), 49.5°C (for OCT-OMC - SLN) and 51.4°C (for OCT-BEMT - SLN), respectively. The narrow of melting range in the case of SLNs is a proof of surfactants presence inside the lipid network that confers a more ordered arrangement. Moreover, by comparing the free SLN with SLN loaded with OCT-OMC and OCT-BEMT, it may be observed that the incorporation of UV absorbers inside the solid lipid matrix has led to a decrease of crystallin arrangement, pointed out by the decrease of endothermal peak intensity.

### Photoprotective effect. *In vitro *determination of SPF

**Figure 6:**
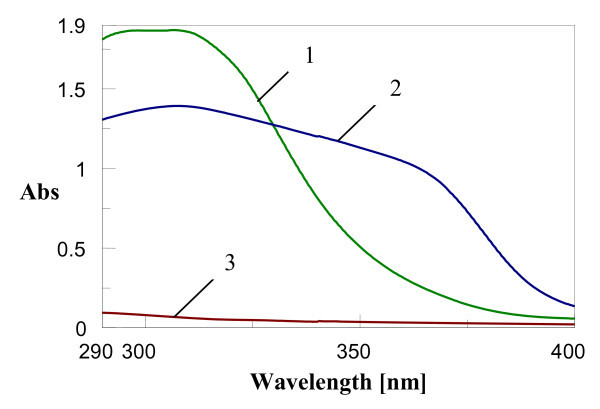
**Wavelength scans of (1) lyophilised OMC-OCT - SLN; (2) lyophilised BEMT-OCT - SLN; (3) empty base cream**.

SPF is the most reliable indicator of the efficacy of sunscreen filters, defined as the sun radiation dose required producing the minimum erythemal dose after application of 2 mg/cm^2 ^of sunscreen on unprotected skin [23]. The UV-Vis spectra of lyophilised SLN containing 8.3% mixture of OCT-OMC and 7.14% OCT-BEMT (referring to the lipid matrix and surfactant composition of SLN) are presented in Figure 6. The protection regions are clear evidenced for both SLN types when compare to the base cream. As expected, due to the BEMT presence, the absorption region of BEMT-OCT is larger than OMC-OCT, this covering a wide UV domain, between 290 and 375 nm. In both prepared sunscreen - SLNs, the encapsulation led to a synergistic UV blocking effect due to the size effect induced by the optimised surfactant composition and lipid matrix which are known to manifest an anti UV-effect [24].

The *in vitro *determination of SPF, based on Diffey method for the empty base cream was SPF = 1, whilst for lyophilised OMC-OCT - SLN and BEMT-OCT - SLN were 19.9 and 19.3, respectively, which assure a good photoprotection, filtering about 95% of UV radiation.

### Photostability behaviour of UV absorbers - SLNs incorporated into a cosmetic carrier

In order to facilitate the spreading onto a synthetic skin, the lyophilised SLNs have been incorporated in an appropriate cosmetic carrier (a base cream) that does not induce the dissolution or aggregation of lipid nanoparticles. The cream formulations have been prepared by dispersing various amounts of lyophilised sunscreen-SLNs in the cream base, so that the final cream formulations contained 0.5, 1.25 and 4.5% sunscreens mixture (w/w), which means less than half of maximum concentration recommended by Food Drug Administration regulations (for OCT is 7.5% and for OMC and BEMT is 10%).

**Figure 7:**
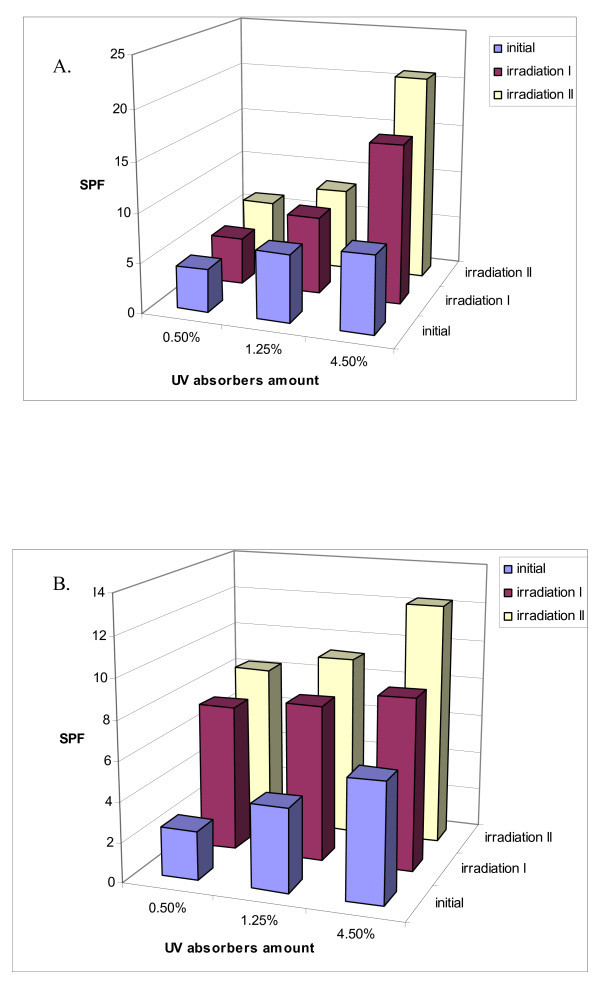
**Effect of irradiation on SPF value for three cream formulations which contain: (a) OCT-OMC - SLN; (b) OCT-BEMT - SLN**.

The composition of the lipid core influences significantly the specific properties of developed formulations. Thus, the UV absorber type loaded into SLN led to different behaviours at irradiation on short time. The effect of irradiation conditions on SPF values of the cosmetic formulations (Figure 7) was examined on wavelength range 290-400 nm, by irradiation in simulated tanning conditions. The irradiation conditions have been chosen to mimic the low energy existent in the middle day (19.5 J/cm^2^) [25]. The results shown in Figure 6 have demonstrated that after UV irradiation, the photoprotective effect has been significantly increased regardless of content of UV absorbers, as comparing with formulations before irradiation. For comparison purpose, the same amount of SLN without UV absorbers has been subjected to UV irradiation, but the initial SPF value of 1.2 has been almost unchanged (SPF after first irradiation period was 1.3 and after second period was 1.2).

Even the BEMT has a broad UV-A and UV-B blocking action, the SPF values for OCT-OMC couple are higher (SPF = 16 after *irradiation I *and 20.8 after *irradiation II*), as comparing to the BEMT-OCT couple (SPF = 8.7 after *irradiation I *and 12.3 after *irradiation II*), for a content of 4.5% molecular sunscreens. Having in view the fact that these UV absorbers manifest a good photostability [26], they do not undergo significant chemical change/photodegradation, allowing them to retain the UV-absorbing capacity [27]. The main reason for this behaviour may be explained based on the structure of UV molecular absorbers, OCT and OMC have double bonds conjugated with carbonyl groups, which upon exposure to UV light undergo isomerisation phenomena and are transformed into keto-enolic form. The BEMT molecule does not present carbonyl groups, thus avoiding such phenomena.

## Conclusion

The encapsulation of both OMC + OCT and OCT + BEMT UV couples into lipid matrices led to average particle size less than 100 nm, with a relatively narrow particle distribution (PI <0.244), using an efficient high shear homogenisation method. All the colloidal systems of nanoparticles have presented zeta potential values less than -50 mV, which assure a high stability of prepared SLNs dispersions.

The crystallisation phenomena of the lipid phase coupled with microscopy images emphasise the presence of the less ordered crystal structure of spherical shape (α-form), whilst avoiding the appearance of undesired perfect crystals of needle shape, characteristic for β modification.

The *in vitro *determination of photoprotection has led to high SPF ratings, with values of 19.9 and 19.3, respectively, for OMC-OCT - SLN and BEMT-OCT - SLN (with 8.3% mixture of OCT-OMC and 7.14% OCT-BEMT), which assure a good photoprotection, filtering about 95% of UV radiation.

The photostability of developed cosmetic formulations based on sunscreen-SLNs has been evaluated by exposure to a photochemical UV irradiation at a low energy. The photoprotection effect after irradiation stage of molecular sunscreens into lipid nanoparticles was observed to be increased more than twofold compared to initial samples. The incorporation of two sunscreen couples into SLN leads to a further advantage - penetration of UV absorbers into the skin is thereby reduced, resulting in a positive effect on the toxicological potential of the UV absorbers. Thus, it is possible to obtain a good photoprotection effect, an improved photostability and a lower allergenic potential using these sunscreen couples, thus demonstrating that UV absorbers-SLNs are promising carrier systems for cosmetic formulations.

## Competing interests

The authors declare that they have no competing interests.

## Authors' contributions

IL conceived of the study, performed the synthesis of the lipid nanoparticles loaded with different UV absorbers, investigate the changes in the crystallinity of the lipid matrix and carried out the TEM analysis. NB carried out the in vitro determination of SPF ratings and the evaluation of absorber couples - lipid nanoparticles photostability by a UV-A and UV-B irradiation study. AMu participed in the synthesis step and in the size distribution evaluation by DLS technique. AMe participated in the drafting of the study and its coordination.
